# Vestibular stimulation-induced facilitation of cervical premotoneuronal systems in humans

**DOI:** 10.1371/journal.pone.0175131

**Published:** 2017-04-07

**Authors:** Shinya Suzuki, Tsuyoshi Nakajima, Shun Irie, Ryohei Ariyasu, Tomoyoshi Komiyama, Yukari Ohki

**Affiliations:** 1 Department of Integrative Physiology, Kyorin University School of Medicine, Mitaka City, Tokyo, Japan; 2 Division of Health and Sports Education, The United Graduate School of Education, Tokyo Gakugei University, Koganei City, Tokyo, Japan; 3 Division of Health and Sports Sciences, Faculty of Education, Chiba University, Chiba City, Chiba, Japan; McGill University Department of Physiology, CANADA

## Abstract

It is unclear how descending inputs from the vestibular system affect the excitability of cervical interneurons in humans. To elucidate this, we investigated the effects of galvanic vestibular stimulation (GVS) on the spatial facilitation of motor-evoked potentials (MEPs) induced by combined pyramidal tract and peripheral nerve stimulation. To assess the spatial facilitation, electromyograms were recorded from the biceps brachii muscles (BB) of healthy subjects. Transcranial magnetic stimulation (TMS) over the contralateral primary motor cortex and electrical stimulation of the ipsilateral ulnar nerve at the wrist were delivered either separately or together, with interstimulus intervals of 10 ms (TMS behind). Anodal/cathodal GVS was randomly delivered with TMS and/or ulnar nerve stimulation. The combination of TMS and ulnar nerve stimulation facilitated BB MEPs significantly more than the algebraic summation of responses induced separately by TMS and ulnar nerve stimulation (i.e., spatial facilitation). MEP facilitation significantly increased when combined stimulation was delivered with GVS (*p* < 0.01). No significant differences were found between anodal and cathodal GVS. Furthermore, single motor unit recordings showed that the short-latency excitatory peak in peri-stimulus time histograms during combined stimulation increased significantly with GVS. The spatial facilitatory effects of combined stimulation with short interstimulus intervals (i.e., 10 ms) indicate that facilitation occurred at the premotoneuronal level in the cervical cord. The present findings therefore suggest that GVS facilitates the cervical interneuron system that integrates inputs from the pyramidal tract and peripheral nerves and excites motoneurons innervating the arm muscles.

## Introduction

Anatomical and electrophysiological studies in animals have suggested that the interneurons (INs) located in the cervical cord integrate the vestibular signals related to altered head position in space and the motor signals related to forelimb movement [1–3]. A variety of INs in the cat cervical cord receive synaptic inputs from vestibular afferents [2, 4]. Based on the anatomical locations of their cell bodies in the grey matter and their caudally projecting axons, some INs are considered propriospinal neurons (PNs) that regulate the vestibular reflexes of the fore- and hind limbs [5]. Furthermore, PNs receive pyramidal tract inputs [6, 7]. Therefore, the vestibular system might communicate with the cervical IN system that conveys outputs from the pyramidal tract to the motoneurons innervating the arm muscles.

To the best of our knowledge, however, comparatively little is known about whether and how vestibular information affects the cervical IN system in humans. Phasic arm movements can be triggered by unexpected head rotations in infants [8]. Even in adults, a fall- or slip-related head movement during standing or walking induces rapid arm movements that might help to prevent head injuries [9–11]. Thus, it is reasonable to hypothesize that descending vestibular inputs modulate the cervical motor system, including the IN networks.

Galvanic vestibular stimulation (GVS) is a noninvasive technique that has been used to study the vestibular system in humans [12–14]. Some studies have demonstrated that GVS elicits reflex responses in voluntarily activated arm muscles [15, 16]. However, no evidence exists that GVS affects the cervical IN systems because the changes observed in ongoing electromyograms (EMGs) recorded after GVS reflect the net postsynaptic effects on motoneuronal pools regardless of the intercalated pathways [17]. To overcome this issue, a spatial facilitation technique may enable us to investigate excitability changes in the presumed IN system in humans [17, 18]. Some research groups have demonstrated that the combined stimulation of the pyramidal tract and peripheral nerves facilitates motor evoked potentials (MEPs) in a variety of arm muscles in humans [19–21]. The effects of the combined stimulation with short inter-stimulus intervals (ISIs) were greater than the summation of the effects of the separate stimuli, which indicated that the facilitation occurred at the premotoneuronal level within the cervical cord [22]. Therefore, these techniques allow us to investigate the vestibular-related influences on the presumed cervical IN system mediating pyramidal tract excitation of arm motoneurons.

The aim of the present study was to investigate whether GVS modulated the spatial facilitatory effects of the combined stimulation of the pyramidal tract and peripheral nerves on arm muscles. The present study postulated that the human vestibular system acts on the cervical IN system that controls arm muscles. The preliminary results of the present study have been presented in abstract form [23].

## Materials and methods

### Subjects

The experiments were conducted on 14 healthy subjects (two females, 22–38 years old) who provided written informed consent. All of the procedures of the present study were approved by the Ethical Committee of the Kyorin University School of Medicine and were in accordance with the Declaration of Helsinki (2013).

The subjects were seated on a chair with a head- and backrest with their eyes open. Their right forearm was rigidly fixed on a platform so that their shoulder and elbow joints were kept at semi-flexed positions (approximately 30° and 130°, respectively).

#### EMG recordings

The surface EMG signals in the right biceps brachii (BB) and first dorsal interosseous (FDI) muscles were recorded with a pair of disposable Ag/AgCl electrodes (Vitrode F-150S, Nihon Kohden Corporation, Tokyo, Japan) using a belly-tendon montage. Before electrode placement, the skin impedance was reduced below 10 kΩ by light abrasions and cleansing of the skin with alcohol. For the BB, the active electrode was then placed over the mid-point of the line between the coracoid process of the scapula and fossa cubit, and the reference electrode was placed on the distal tendon. For the FDI, the active electrode was placed over the mid-point of the muscle belly, and the reference electrode was placed on the head of the second metacarpal. The surface EMG signals were amplified (1,000 times), bandpass filtered (15–3,000 Hz) with an amplifier (AB-611J, Nihon Kohden Corporation), and sampled at 6 kHz with an A/D converter (Power 1401, Cambridge Electronic Design, Ltd., Cambridge, UK). In some experiments, the activity of a single motor unit (MU) was recorded with a bipolar concentric needle electrode (NM-030T, Nihon Kohden Corporation). The needle was inserted so that the tip was close to the neuromuscular junction, which was located close to the location of the innervation point (cf., [24]). The activities of single MUs were carefully monitored on-line by extracting the individual MU spikes based on their shapes with a spike discriminator (Alpha Spike Detector; Alpha Omega Engineering, Nazareth, Israel) and by displaying them on a monitor. The intramuscular EMG signals were amplified (10,000 times), band-pass filtered (15–10,000 Hz), and sampled at 20 kHz.

For normalization of the EMG amplitudes, the maximum voluntary activation level of the BB was determined at the beginning of each experiment, as described below. The subjects were asked to perform a maximum voluntary isometric elbow flexion of their right arm for ~3 s. This was repeated three times with 30-s inter-trial intervals. The maximum values of the mean amplitudes of the rectified BB EMGs in 1-s periods in the middle of each 3 s trial were calculated for each trial. The average amplitude across the trials was called the EMGmax.

### Transcranial magnetic stimulation (TMS)

TMS, which consisted of a single monophasic pulse, was delivered over the left primary motor cortex with a magnetic stimulator (Magstim 200, The Magstim Company, Ltd., Whitland, UK). A figure-of-eight-shaped coil (70 mm diameter) was held over the arm area of the motor cortex on the scalp to induce anteromedial current flow in the brain [25]. The optimal position and direction of the coil were carefully selected so that an MEP was evoked in the relaxed BB. The coil was operated by two skilled experimenters. The active motor threshold (AMT) for TMS was determined as the minimum stimulus intensity at which a MEP with a peak-to-peak amplitude over 100 μV was evoked in five of 10 consecutive trials under tonic contraction of the BB (~3% of the EMGmax).

### Electrical peripheral nerve stimulation

Electrical stimulation with a single rectangular pulse for 200 μs was delivered to the right ulnar nerve at the wrist with a pulse regulating system (SEN-8203; Nihon Kohden Corporation) and isolator (SS-104J; Nihon Kohden Corporation). A pair of surface electrodes (Vitrode F-150S, Nihon Kohden Corporation) was carefully placed on the optimal site for evoking a larger direct motor (M-) wave in the FDI at the lowest stimulus intensity. The stimulus intensity of the ulnar nerve stimulation was expressed as multiplies of the motor threshold (MT) of the FDI. The MT was defined as the minimum stimulus intensity that evoked an M-wave and twitch, which was confirmed by tendon palpation.

### GVS

For GVS, electrical stimulation with a single rectangular pulse for 1 s was delivered from another constant-current isolator unit (SS-104J; Nihon Kohden Corporation). A pair of disposable surface electrodes (Vitrode F-150S, Nihon Kohden Corporation) was placed over the bilateral mastoid processes for bipolar stimulation [15, 16, 26, 27]. We used two electrode montages for GVS: anodal and cathodal GVS (e.g., anodal GVS indicated that the right electrode was the anode). All of the subjects confirmed verbally that they perceived the roll of their head toward the anodal side during GVS [28, 29]. The stimulus intensity of the GVS was expressed as multiplies of the threshold of the roll perception (i.e., perceptual threshold: PT). The PT ranged from 0.7 to 1.0 mA.

### Experimental tasks

**Fig 1:**
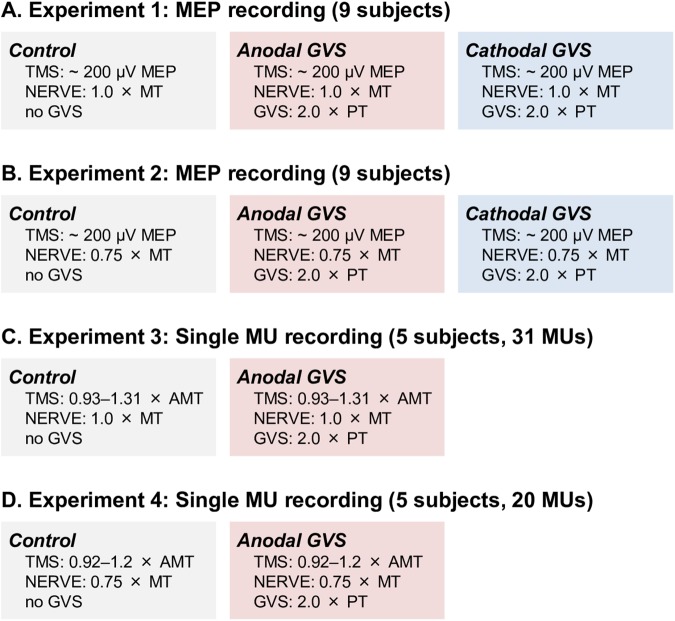
Experimental tasks in the present study. **A**–**D** show the number of subjects, number of test sequences, and stimulus conditions in each experiment. Each block indicates the test sequence used to assess the spatial facilitation effects. The stimulus intensities of the transcranial magnetic stimulation (TMS), ulnar nerve stimulation (NERVE), and galvanic vestibular stimulation (GVS) are indicated within the block. The order of the test sequences was randomly selected. MEP: motor-evoked potential, MT: motor threshold of the first dorsal interosseous muscle, PT: perceptual threshold of GVS-induced head sway, MU: motor unit, AMT: active motor threshold of the biceps brachii muscle.

In the present study, four independent experiments were sequentially conducted in different days (experiments 1–4 in Fig 1). The 14 subjects participated in either one or more than two experiments. First, 9 of the 14 subjects participated in the MEP-recording experiment involving ulnar nerve stimulation at 1.0 × MT (experiment 1, Fig 1A). Second, 9 of the 14 subjects, including 6 subjects who participated in experiment 1, participated in the additional control MEP-recording experiment involving ulnar nerve stimulation at 0.75 × MT (experiment 2, Fig 1B). Third, 5 of the 14 subjects, including 4 subjects who participated in experiment 1, were included in the MU-recording experiment involving ulnar nerve stimulation at 1.0 × MT (experiment 3, Fig 1C). Lastly, 5 of the 14 subjects, including 4 subjects who participated in experiment 1, were included in the MU-recording experiment involving ulnar nerve stimulation at 0.75 × MT (experiment 4, Fig 1D).

### Experimental procedures

**Fig 2:**
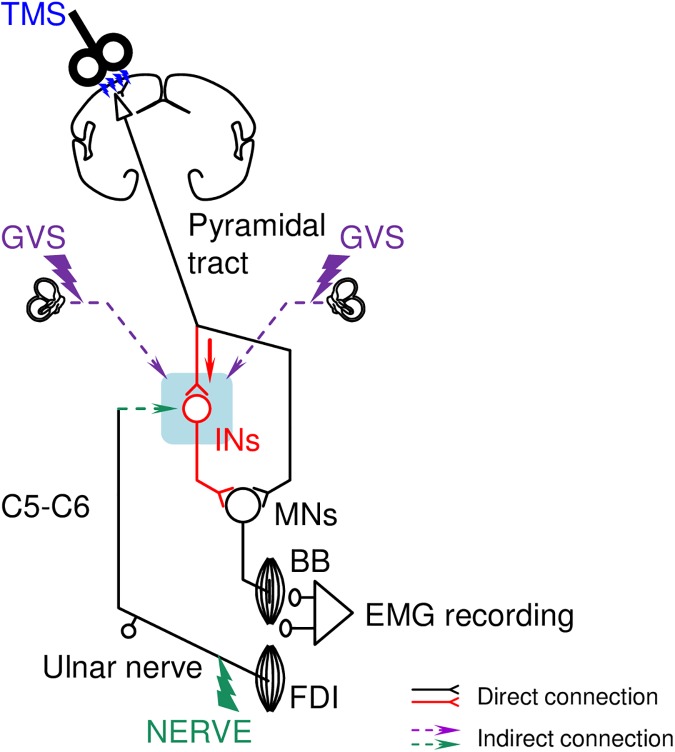
Schematic of the methodology and potential premotoneuronal pathways in the cervical cord. The wiring pattern is oversimplified for better understanding. Black and red solid lines represent direct (monosynaptic) connections, whereas purple and green dashed lines represent indirect (non-monosynaptic) connections no matter whether its effect is facilitatory or inhibitory. The pyramidal tract volleys (a red arrow) that are produced by transcranial magnetic stimulation (TMS) over the contralateral motor cortex and the afferent volleys (a green arrow) that are produced by electrical stimulation of the ipsilateral ulnar nerve (NERVE) at the wrist converge onto a common cervical interneuron (IN) that projects to motoneurons (MNs) of the biceps brachii (BB) muscle, which results in extra facilitation of motor-evoked potentials (MEPs) in the BB. The TMS and NERVE are timed so that the pyramidal tract and afferent volleys simultaneously arrive at the upper cervical cord. The inputs produced by galvanic vestibular stimulation (GVS) through the bilateral mastoid processes also converge on the IN pool. FDI: first dorsal interosseous muscle, EMG: electromyogram.

The test sequences consisted of three types of stimulus trials: (1) separate ulnar nerve stimulation, (2) separate TMS, and (3) combined ulnar nerve stimulation and TMS. For the combined stimulation, a 10-ms inter-stimulus interval (ISI) was used, with ulnar nerve stimulation delivered before the TMS. The rationale for the use of this ISI was based on the findings of our preliminary experiments on 5 subjects that showed that the combination of TMS and ulnar nerve stimulation with a 10-ms ISI efficiently facilitated the MEPs compared with stimulations with other ISIs (i.e., 6, 7, 7.5, 8, 9, 11, 12, and 15-ms ISIs) in all of the subjects. Based on the results of previous studies, the conduction time was estimated such that the afferent time from the wrist to the upper cervical cord (green arrow in Fig 2) and the efferent time from the motor cortex to the cervical cord (red arrow in Fig 2) were ~14 ms [30, 31] and 3.6–4 ms [32], respectively. Thus, a 10-ms ISI, together with analyses of short latency effects starting around MEP onset, allowed us to investigate the effects of the simultaneous convergence of inputs from the ulnar nerve at the wrist level and the pyramidal tract onto cervical INs. The three types of stimulus trials were randomly delivered with intertrial intervals of 4–6 s in the MEP-recording experiments and 2–3 s in the MU-recording experiments. The number of stimuli in each stimulus condition was 10 stimuli for the MEP-recording experiments and 50 stimuli for the MU-recording experiments. In the MEP-recording experiments, three test sequences were performed under different GVS conditions, i.e., without (control) and with anodal or cathodal GVS. Because no significant differences were found between anodal and cathodal GVS, we focused on the control and anodal GVS conditions in the MU-recording experiments in order to complete the recordings from the same MU in minimal time. In the GVS conditions, the TMS was delivered 0.5 s after the beginning of the GVS pulse. The long ISI in the GVS was selected in order to avoid any confounding ON/OFF effects of the GVS itself on the background EMG activity [15, 16, 33]. The orders of the test sequences were randomized.

During the test sequences, the subjects were asked to perform a tonic voluntary elbow flexion. In the MEP-recording experiments, the contraction level was set at ~ 3% of the EMGmax. In the MU-recording experiments, the subjects were instructed to voluntarily activate a single MU at ~10 Hz. To maintain the contraction at the desired level, visual and/or auditory feedback of the BB EMG were continuously given.

For each subject, the TMS intensity was predetermined so that MEPs with peak-to-peak amplitudes of ~200 μV were evoked [amplitude (mean ± standard error of the mean): 227.6 ± 1.05 μV; stimulus intensity: 1.1–1.36 × AMT]. The test MEP sizes did not differ across the GVS conditions and experiments when they were normalized to the peak-to-peak amplitude of the BB maximum M-wave (Mmax), which was evoked by supramaximal electrical stimulation of the brachial plexus at the supraclavicular fossa [experiment 1: amplitude (mean ± standard error of the mean), 1.1 ± 0.066% of the Mmax; experiment 2: amplitude (mean ± standard error of the mean), 0.945 ± 0.061% of the Mmax; two-way analysis of variance (ANOVA): GVS condition, *F*_2, 32_ = 1.642, *p* = 0.209; experiment, *F*_1, 16_ = 0.977, *p* = 0.338; GVS condition × experiment, *F*_2, 32_ = 1.05, *p* = 0.363]. In the MU-recording experiments, a slightly weak intensity (0.92–1.32 × AMT) was used in order to prevent additional recruitment of non-target MUs or compound muscle action potentials. In the experiments with TMS intensities set above the AMTs, it was carefully ascertained that the TMS did not evoke compound MEPs in the intramuscular EMG during the MU recordings. In addition, we sometimes used lower intensities (i.e., below AMT) in case stronger stimulations (i.e., above AMT) induced activities in other MUs that were indistinguishable from the target MU activities. However, even in these cases, compound MEPs with small amplitudes were evoked in the surface EMG [amplitude (mean ± standard error of the mean): 72 ± 7.3 μV]. Thus, it is possible that the pyramidal tract neurons were activated at least in part by TMS and that these volleys reached the cervical cord. The stimulus intensity of the ulnar nerve stimulation was typically set at 1.0 × MT [19]. In the separate experiments performed on different subjects (n = 9), the spatial facilitation effects on the MEPs were tested with ulnar nerve stimulation at 0.75 × MT [20]. This experiment was conducted in order to determine the contribution of large-diameter afferent fibers (e.g., group Ia muscle spindle afferents) by reducing the activation of afferents with relatively small diameters (e.g., group II cutaneous afferents), and prevent the facilitation of the MEPs being truncated by inhibition [19, 20]. The GVS intensity was set at 2.0 × PT. We confirmed that GVS by itself did not evoke any motor responses in the BB EMG by cervical root stimulation.

### Data analyses

#### MEPs

In order to evaluate the magnitude modulation of the MEPs, the full-wave rectified EMG signals in the same stimulus type were sorted together and averaged across 10 trials. The mean background EMG amplitude during a 50-ms period before TMS in each full-wave rectified and averaged EMG was subtracted from each averaged trace. The MEP area was then calculated between the signal and zero over the MEP duration. The analysis window for the area calculation started and ended when the EMG exceeded and fell below 1 standard deviation (SD) of the background EMG during the prestimulus period (50 ms), respectively. Because the amplitudes of the EMG responses that were evoked by ulnar nerve stimulation were usually too small to be determined (below 2 SD of the background EMG), the same analysis window that was used in the trials with combined stimulation was used. The summation (SUM) of the MEP areas in the trials with separate stimuli was then calculated. For intersubject comparisons of the spatial facilitatory effect, the MEP area in the combined stimulation trial was normalized by the SUM.

#### Peri-stimulus time histograms (PSTHs)

In order to determine the changes that occurred in the firing probability of single MUs in response to the stimuli, we constructed PSTHs from spike trains of voluntarily activated single MUs [34, 35]. The data for approximately 50 trials per stimulus type were analyzed [19, 20]. Individual MU spikes were discriminated on-line and converted into transistor-transistor logic pulse events. After the events were sorted together for each stimulus condition, the PSTHs were constructed by counting the events in every 0.5-ms bin after the trigger [20]. The mean event counts over a 50-ms prestimulus period (background counts) were subtracted from the event counts in each bin. The onset latency of the TMS-induced excitation in the PSTH was the time when the event counts consecutively exceeded + 1 SD of the background counts for 1 ms. Differential PSTHs were constructed by subtracting the summation of the PSTHs for separate TMS and ulnar nerve stimulation from the PSTH for combined stimulation [22]. The mean event counts in the differential PSTH were then calculated during a defined analysis window that started 1 ms after the onset latency. This period was chosen for analysis as the initial 1-ms bins of the TMS-induced PSTH peak were less affected by peripheral nerve stimulation or GVS (see below and [22]). The analysis window was 1-ms long in order to include the first wave component of several successive descending waves [32]. The event counts were normalized by the number of triggers [22].

### Statistics

A two-way repeated-measures ANOVA was performed with three within-factor levels (control, anodal, and cathodal GVS conditions) and two levels (combined stimulation and algebraic summation). When the *F*-value was significant, a Bonferroni test was performed to detect significant differences in all of the possible pair-wise comparisons for each variable (EMG area and prestimulus background EMG). In the PSTH group data, paired *t*-tests (control vs. GVS conditions) were used. Furthermore, in order to clarify whether the number of subjects was adequate for each statistical test, a power analysis was performed with G*power (version 3.1.9.2, Heinrich Heine University Düsseldorf, Düsseldorf, Germany, [36]). The effect size indexes were calculated with Cohen’s *d* values for the pairwise comparisons and *f* values for ANOVAs [37]. The statistical power (1 - *β*) was then computed. The level of statistical significance was set at *p* < 0.05, and the level of acceptable statistical power was set at 0.8 [38]. The group data are presented as mean ± standard error of the mean unless otherwise noted.

## Results

### Effects of GVS on ulnar nerve-induced MEP facilitation

**Fig 3:**
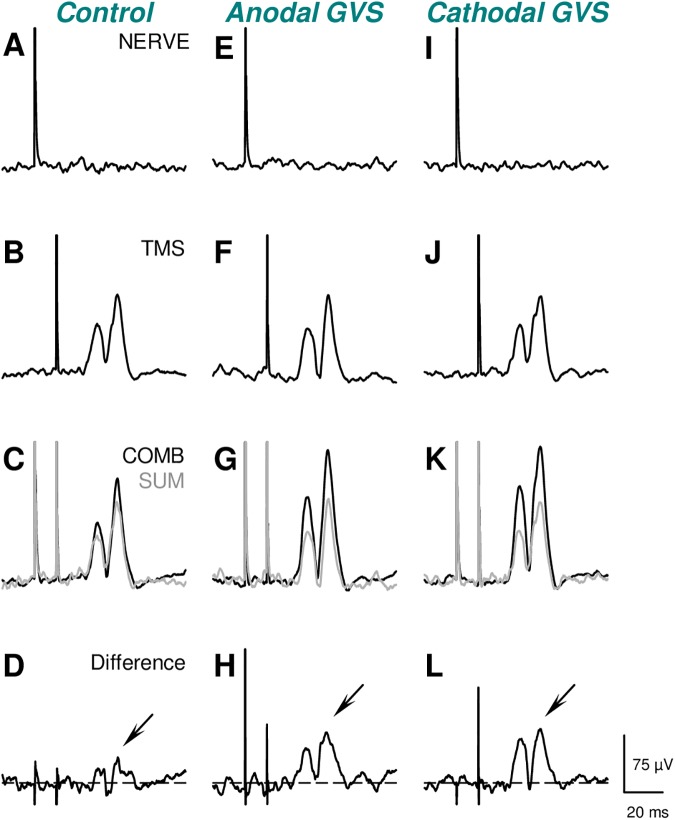
The effects of galvanic vestibular stimulation (GVS) on ulnar nerve-induced facilitation of motor-evoked potentials in a single subject. Full-wave rectified and averaged electromyograms (EMGs) in the biceps brachii (BB) muscle after separate ulnar nerve stimulation (NERVE) at 1.0 × the motor threshold of the first dorsal interosseous muscle (**A**, **E**, **I**), separate transcranial magnetic stimulation (TMS) over the contralateral primary motor cortex at 1.1 × the active motor threshold of the BB (**B**, **F**, **J**), and the combination (COMB) of NERVE and TMS (**C**, **G**, **K**). These waveforms were obtained without GVS (left panels), during anodal GVS (middle panels), and during cathodal GVS (right panels) at 2.0 × the perceptual threshold of the head sway. The grey waveforms in **C**, **G**, and **K** represent the summation (SUM) of the averaged EMG waveforms after separate TMS and NERVE. The waveforms in **D**, **H**, and **L** represent the COMB waveforms with the SUM waveforms subtracted.

Fig 3 illustrates the effects of GVS on the ulnar nerve-conditioned BB MEPs in a single subject. In the control condition, TMS with ulnar nerve stimulation resulted in facilitation of the MEPs (Fig 3C) compared with TMS alone (Fig 3B). Importantly, the facilitatory effects of the combined stimulation were larger than the summation of the effects of the separate stimuli (i.e., TMS alone and ulnar nerve stimulation alone) (grey waveform in Fig 3C), which indicated extra-facilitation effects (downward arrow in Fig 3D). The facilitatory effects of the combined stimulation were markedly augmented in both the anodal and cathodal GVS conditions with respect to the control condition (Fig 3G and 3K, see also 3H and 3L).

**Fig 4:**
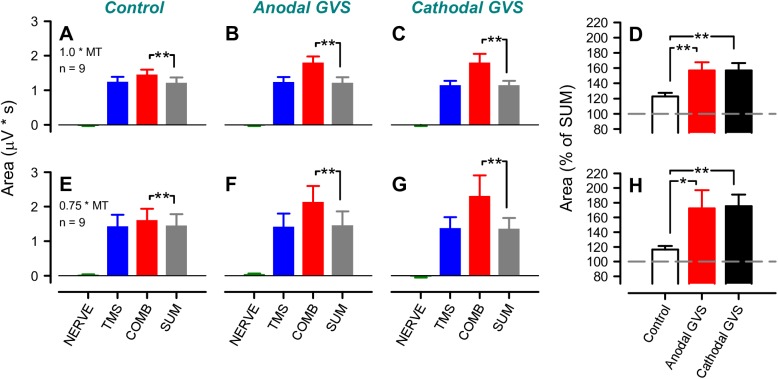
The pooled data for the effects of galvanic vestibular stimulation (GVS) on the ulnar nerve-induced facilitation of motor-evoked potentials (MEPs) across subjects. The pooled data for the mean areas of the MEPs in the biceps brachii muscle after separate ulnar nerve stimulation (NERVE), separate transcranial magnetic stimulation (TMS) over the contralateral primary motor cortex, and the combination (COMB) of NERVE and TMS in the control (**A**, **E**), anodal GVS (**B**, **F**), and cathodal GVS conditions (**C**, **G**). **D** and **H** show the average of the amount of spatial facilitation in the control and GVS conditions. The areas of the MEPs after the combined stimuli were normalized by the summation of the areas of the MEPs recorded after separate TMS and NERVE. **A**–**D** and **E**–**H** illustrate the data from experiments involving ulnar nerve stimulation at 1.0 and 0.75 × the motor threshold (MT) of the first dorsal interosseous muscle, respectively. The error bars represent standard errors of the mean. The asterisks indicate a statistically significant difference between conditions (*p < 0.05, **p < 0.01).

Fig 4 shows the grouped data for the MEP responses that were evoked by the combined stimulation with and without GVS across all of the subjects. The MEP areas following combined stimulation with ulnar nerve stimulation of 1.0 × MT were significantly larger than the summed MEP areas of TMS and ulnar nerve stimulation. Furthermore, the MEP areas were larger in the GVS conditions than in the control condition (Two-way ANOVA: GVS condition, *F*_2, 16_ = 8.14, *f* = 0.623, *p* < 0.01, 1 - *β* = 0.968; stimulus trial, *F*_1, 8_ = 24.6, *f* = 2.576, *p* < 0.01, 1 - *β* = 0.999; GVS condition × stimulus trial, *F*_2, 16_ = 8.15, *f* = 1.07, *p* < 0.01, 1 - *β* = 0.999; Bonferroni test: *p* < 0.01, Fig 4A–4C). Thus, the amounts of extra-facilitation in both GVS conditions were significantly larger than that in the control condition (One-way ANOVA: *F*_2, 16_ = 8.15, *f* = 1.01, *p* < 0.01, 1 - *β* = 0.999; Bonferroni test: *p* < 0.01, Fig 4D). Furthermore, the amounts of facilitation did not differ between the two GVS conditions (Bonferroni test: *p* = 0.998, Fig 4D). With ulnar nerve stimulation at 0.75 × MT, the amount of ulnar-induced MEP facilitation in the GVS conditions was larger than that in the control condition (one-way ANOVA: *F*_2, 16_ = 7.71, *f* = 0.982, *p* < 0.01, 1 - *β* = 0.999; Bonferroni test: control vs. anodal GVS, *p* < 0.05; control vs. cathodal GVS, *p* < 0.01; anodal vs. cathodal GVS, *p* = 0.99, Fig 4H).

On the other hand, the response areas under separate stimuli (i.e., TMS alone or ulnar nerve stimulation alone) were not significantly different among the three conditions (1.0 × MT intensity: TMS alone, *F*_2, 16_ = 0.891, *f* = 0.334, *p* = 0.43, 1 - *β* = 0.502; ulnar nerve stimulation alone, *F*_2, 16_ = 0.306, *f* = 0.196, *p* = 0.741, 1 - *β* = 0.199; 0.75 × MT intensity: TMS alone, *F*_2, 16_ = 0.289, *f* = 0.19, *p* = 0.753, 1 - *β* = 0.19; ulnar nerve stimulation alone, *F*_2, 16_ = 1.335, *f* = 0.408, *p* = 0.291, 1 - *β* = 0.684). The mean amplitudes of the prestimulus EMGs were constant across conditions and stimulus trial types (1.0 × MT intensity: GVS condition, *F*_2, 16_ = 0.603, *f* = 0.41, *p* = 0.559, 1 - *β* = 0.686; stimulus trial, *F*_2, 16_ = 1.46, *f* = 0.332, *p* = 0.262, 1 - *β* = 0.498; GVS condition × stimulus trial, *F*_4, 32_ = 0.275, *f* = 0.205, *p* = 0.892, 1 - *β* = 0.143; 0.75 × MT intensity: GVS condition, *F*_2, 16_ = 1.187, *f* = 0.385, *p* = 0.33, 1 - *β* = 0.63; stimulus trial, *F*_2, 16_ = 1.397, *f* = 0.418, *p* = 0.276, 1 - *β* = 0.705; GVS condition × stimulus trial, *F*_4, 32_ = 1.407, *f* = 0.419, *p* = 0.254, 1 - *β* = 0.507).

### Effects of GVS on the firing probability of single MUs after combined TMS and ulnar nerve stimulation

**Fig 5:**
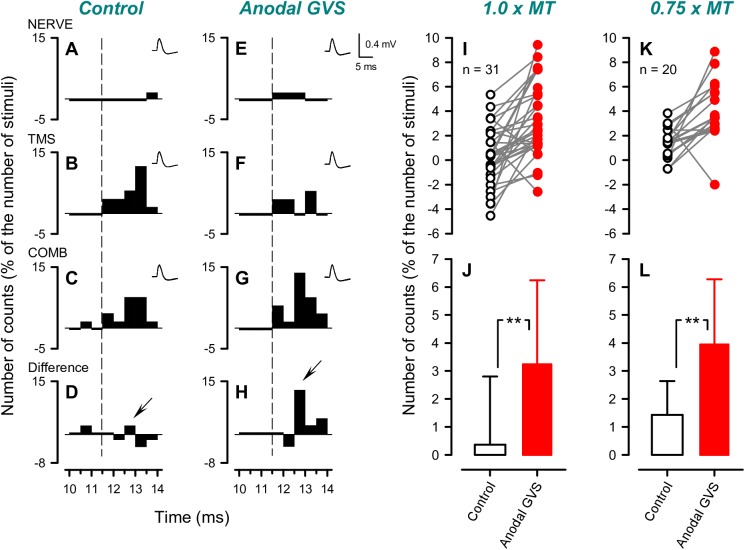
The effects of galvanic vestibular stimulation (GVS) on the firing probability of single motor units (MUs) after combined motor cortex and ulnar nerve stimulation. **A**–**H** show the peristimulus time histograms (PSTHs) of a single MU in the biceps brachii (BB) muscle after separate ulnar nerve stimulation (NERVE) at 1.0 × motor threshold (MT) of the first dorsal interosseous muscle (**A**, **E**), separate transcranial magnetic stimulation (TMS) (**B**, **F**) over the contralateral primary motor cortex at 1.25 × active motor threshold of the BB, and combined stimulation (COMB) (**C**, **G**) in the control (**A**–**D**) and anodal GVS conditions (**E**–**H**). Each PSTH was obtained after 50 stimuli. The counts in these PSTHs were subtracted by the mean counts during a 50 ms prestimulus period. **D** and **H** show differential PSTHs after subtraction of the summed PSTHs after separate stimuli from the PSTHs of the COMB. The number of counts in each bin was normalized by the number of triggers. The vertical dashed line represents the onset of the excitatory peak in the PSTH after separate TMS. The superimposed waveforms in the upper right corner of each PSTH show the MU action potentials (n = 50) obtained from each stimulus trial. **I**–**L** indicate the peak counts of the MU firings in the differential PSTHs in the control and anodal GVS conditions obtained from 31 MUs that were investigated with the NERVE set at 1.0 × MT (**I**, **J**) and 20 MUs that were investigated with the NERVE set at 0.75 × MT (**K**, **L**). The error bars represent 1 standard deviation. The analysis window was set at a predefined period (1.0 ms duration) that started 1.0 ms after the onset of the TMS-induced excitatory peak in the PSTH. **p < 0.01.

Fig 5A–5H illustrate the PSTHs of a single MU in the control and anodal GVS conditions. In the control condition, combined TMS (1.25 × AMT) and ulnar nerve stimulation (1.0 × MT) produced a slight increase in firing probability at a short latency (downward arrow in Fig 5D). Interestingly, the changes became markedly apparent in the GVS condition (downward arrow in Fig 5H). This facilitation started 1 ms after the onset of the excitation that was induced by separate TMS (onset latency: 11.5 ms in both conditions, shown with vertical dotted lines). The mean (± SD) background firing frequency of the MU was 9.71 ± 1.41 Hz. Fig 3I and 3J show the group peak counts in the differential PSTHs with ulnar nerve stimulation at 1.0 × MT obtained from all successfully recorded MUs (n = 31). In 27 of the 31 MUs (87.1%), the facilitatory effects induced by combined stimulation 1 ms after the onset of TMS-induced excitation were larger in the anodal GVS condition than in the control condition (Fig 5I). The firing probabilities of the two conditions differed significantly (*d* = 0.854, *p* < 0.01, 1 - *β* = 0.996, Fig 5J). In contrast, the firing probability within the first 1-ms bins of the TMS-induced peak was not affected by GVS (*d* = 0.079, *p* = 0.663, 1 - *β* = 0.071).

Fig 5K and 5L show the population data for the peak counts in the differential PSTHs with ulnar nerve stimulation at 0.75 × MT in 20 MUs. In 18 of the 20 MUs (90%), the delivery of the combination of TMS with ulnar nerve stimulation and anodal GVS increased the extra facilitation in the PSTH peak. The differences in the extra counts between the control and anodal GVS conditions were statistically significant (*d* = 0.954, *p* < 0.01, 1 - *β* = 0.981, Fig 5L) in the time window of 1 ms after onset latency but not within the first 1-ms bin of the TMS-induced peaks (*d* = 0.336, *p* = 0.149, 1 - *β* = 0.298).

The latencies did not differ between the two conditions (1.0 × MT intensity, *d* = 0.01, *p* = 0.999, 1 - *β* = 0.05; 0.75 × MT intensity, *d* = 0.087, *p* = 0.705, 1 - *β* = 0.066). The frequencies of the background firing in the MUs did not change across conditions and stimulus trials (two-way ANOVA: 1.0 × MT intensity, GVS condition, *F*_1, 30_ = 3.508, *f* = 0.341, *p* = 0.071, 1 - *β* = 0.439; stimulus trial, *F*_2, 60_ = 0.41, *f* = 0.117, *p* = 0.666, 1 - *β* = 0.099; GVS condition × stimulus trial interaction, *F*_2, 60_ = 0.951, *f* = 0.178, *p* = 0.392, 1 - *β* = 0.108; 0.75 × MT intensity, GVS condition, *F*_1, 19_ = 1.979, *f* = 0.323, *p* = 0.176, 1 - *β* = 0.399; stimulus trial, *F*_2, 38_ = 1.189, *f* = 0.25, *p* = 0.315, 1 - *β* = 0.303; GVS condition × stimulus trial interaction, *F*_2, 38_ = 0.426, *f* = 0.15, *p* = 0.656, 1 - *β* = 0.09).

## Discussion

The present study demonstrated that GVS significantly increased the ulnar-induced facilitation of MEPs in the BB. Moreover, we substantiated that GVS explicitly increased the effects of the combined stimulation on the MU firing probability with recordings of single MUs. The increase in the firing probability was consistently seen ~ 1 ms after the onset of TMS-induced excitation in most MUs.

### Methodological considerations

In the control condition, the effects of ulnar nerve stimulation were too weak for detection of any reflex components in the rectified and averaged EMGs (see Fig 3A, 3E and 3I). Despite that, we found significant facilitation of MEPs following TMS with ulnar nerve stimulation. Furthermore, this facilitation was larger than that estimated by the summation of the responses to separate TMS and ulnar nerve stimulation in all of the subjects. Thus, the extra-facilitation effects are unlikely simple summation effects at the BB motoneuron pool that arise from individual pathways from the inputs evoked by pyramidal tract and ulnar nerve stimulation [17].

Furthermore, convergence of the afferent volleys with corticospinal volleys might occur at subcortical, possibly cervical levels. We used a 10-ms ISI between TMS and ulnar nerve stimulation at the wrist. Considering the conduction velocity (54.3–83.3 m/s) of the ulnar nerve in humans [39, 40] and the latency of the first cortical component (N20) in somatosensory-evoked potentials following ulnar nerve stimulation at the wrist [41], the afferent volleys do not arrive at the cortex when the TMS is delivered to the motor cortex. Conversely, pyramidal volleys arrive at the upper cervical level 3.6–4 ms after stimulation of the motor cortex [32]. Given the latency of the upper cervical/cuneate nucleus component (N13; latency ~14 ms) in somatosensory-evoked potentials [30, 31], a plausible interpretation of the current findings is that the extra-facilitation effects of the combined stimuli were mediated by a shared IN system in the cervical cord that receives both pyramidal tract and peripheral nerve inputs, as has been described by Pierrot-Deseilligny and his co-workers [42].

The selection of the target muscle in the present study might be relevant for investigations of the presumed cervical IN system. In humans, strong facilitation effects mediated by the presumed cervical IN system have been found in physiological flexors of the upper limb, including the BB, but not in intrinsic hand muscles (e.g., FDI) [20, 43, 44]. Further studies are needed to clarify the GVS-induced modification of the effects of combined stimulation on other muscles.

### Possible neural mechanisms of GVS effects on ulnar nerve-induced corticospinal facilitation

As for the surface EMG recordings, it is likely that the subthreshold excitation of motoneurons by each stimulus input could also be generated by spatial facilitation effects in the motoneuron pool themselves following simultaneous combined inputs, even if the EMG responses induced by the separate input are almost negligible [45, 46]. Therefore, we explored the modulation of the firing probability of single MUs in PSTHs following combined stimulation with and without GVS. By using this PSTH technique, we can avoid potential confounds due to unobservable subthreshold motoneuronal excitation in the pool [19, 20, 47]. We found that the initial part (1.0 ms from the onset of excitation, presumably from monosynaptic contribution) of the TMS-induced excitation was not modulated by ulnar nerve stimulation with and without GVS (Fig 5). However, the MU counts between 1.0 and 2.0 ms after the excitation onset were facilitated with ulnar nerve stimulation in almost all MUs, and facilitation was higher with GVS. These delays were assumed to be longer than the latency variability of the firing probability of the motoneurons, which is related to the fluctuations of the amplitudes of postsynaptic potentials (<0.35 ms [48]). Considering the synaptic delay within corticospinal pathways [6], these results suggested that GVS volleys predominantly affected di- or oligosynaptic pyramidal tract excitation that was mediated by the presumed premotoneuronal pathways rather than monosynaptic excitation [20, 22].

When a relatively high stimulation intensity of the peripheral nerve (i.e., 1.0 × MT) is used, the peripheral nerve-induced facilitation of the corticospinal excitation is truncated by inhibition of the cervical cord [19, 20]. In fact, the extra counts in the peaks of the differential PSTHs were relatively small in the control condition when the ulnar nerve stimulation was set at 1.0 × MT in this study (Fig 5D). Therefore, the facilitation effects were further investigated with rather weak ulnar nerve stimulation (i.e., 0.75 × MT), which has been assumed to be optimal for observing facilitation [20]. As a result, the extra facilitation effect in the control condition became clearer in the PSTHs, and GVS increased the extra facilitation (see Figs 4 and 5), which was consistent with the results when the ulnar nerve stimulation was 1.0 × MT. Once again, these GVS effects under the 1.0 × MT intensity might be partly explained by the reduction of the presumed inhibitory effects in the cervical cord [19, 49]. Further investigations are needed to elucidate this finding.

A plausible source of the GVS-induced effects is afferent inputs arising from the labyrinth [13]. Studies of animal preparations have shown that GVS modulates the spontaneous discharge of vestibular afferent fibers [50, 51]. The results of studies on patients who underwent vestibular nerve dissections also support the involvement of peripheral vestibular afferents in GVS-induced responses [12, 27]. In the present investigation, we confirmed that all of the subjects perceived the head roll during the GVS, which suggested that the effects of the GVS in the present study predominantly originated from vestibular afferents, regardless of their origin (i.e., otolith organs and/or semicircular canals) [28, 52–54].

Several central pathways might mediate the GVS effects on the presumed cervical premotoneuronal INs. As observed in cats and monkeys, the descending brainstem pathways (e.g., vestibulospinal- and/or reticulospinal tracts) that receive inputs from primary vestibular afferents and project onto cervical neurons might mediate the vestibular effects [1, 55, 56]. The long ISI that was used for GVS (i.e., 0.5 s after the beginning of the GVS pulse) in the present study allowed the GVS enough time to activate the thalamo-cortico-sub-cortical loops [57, 58], which would have influenced the cortical excitability and, thus, MEP size. However, GVS by itself did not modify the MEP size (Fig 3B, 3F and 3J). In addition, the extra facilitation was not observed in the initial 1-ms bins of the TMS-induced peak in the PSTH (Fig 5B and 5F). These findings suggest that direct monosynaptic pyramidal tract inputs are not affected by GVS at the ISI investigated. In taking into account the neuronal mechanisms that induce the spatial facilitation, the present findings favored the explanation that the GVS affected the shared cervical IN system that receives both pyramidal tract and peripheral nerve inputs.

In the current study, anodal and cathodal GVS similarly affected ulnar nerve-induced MEP facilitation. These results were somewhat confusing because vestibular reflex patterns in forelimb muscles are reversed between left/right vestibular stimulation [59–61]. One plausible explanation is that the findings of the present study were strongly affected by the methodology (e.g., head/body positions and eyes open/closed) and stimulus procedures (e.g., placement of the reference electrode, ISIs in the test) [16, 26, 62–64]. Similar results of a lack of polarity dependence in the lower limb have been reported in investigations of these effects in the sitting position (as in the present study) [26, 65]. Furthermore, the present results might be accounted for by the possibility that the GVS activates systems that bilaterally innervate presumed spinal interneurons [66, 67]. In humans, the non-specific reflex-facilitation that was induced by GVS showed a similar time course as auditory startle reflexes, which suggested that the GVS-induced effects were partly mediated by the reticulospinal system [26]. Taking these results into account, the present findings suggested that GVS activated bilateral projecting systems, such as the vestibulospinal and/or reticulospinal pathways, through vestibular afferents, which presumably facilitated cervical premotoneurons in a polarity-nonspecific manner (see Fig 2A). However, these details of these mechanisms remain unclear.

The findings of the present study are potentially relevant to the presumed vestibulo-motor regulation of the arm movement [68]. In fact, rapid arm responses can be triggered by fall- or slip-related head movements (e.g., inputs arising from vestibular afferents), which are assumed to contribute to counterbalance and protective reactions [9–11, 69]. In the present study, human vestibular stimulation facilitated the cervical IN system and then excited the arm muscles. Thus, vestibular-related processing in the motor system, including the cervical IN networks, might be helpful for rapid regulation and/or stabilization of the arms in response to multidirectional head movements [68]. Further investigations of functional situations are required to support this assumption.

### Limitations

This study had a number of limitations. One limitation was the use of a single ISI between the ulnar nerve stimulation and TMS (i.e., 10 ms). In other words, testing with a range of ISIs might better individualize the stimulus timings and account for the afferent/efferent conduction times from the stimulus site (ulnar nerve/motor cortex) to the cervical cord in each subject [70]. However, marked MEP facilitation was observed with a 10-ms ISI in all of the investigated subjects when several ISIs were tested in the preliminary stage (n = 5, Suzuki, Nakajima, Komiyama, and Ohki, unpublished observations). Thus, it is likely that the timing was within a range that was reasonable for investigating the convergence effects of the pyramidal tract and peripheral nerve inputs onto the presumed cervical INs.

Another limitation of the present study was that a sample size calculation was not performed before the experiments were conducted. However, in all of the tests with statistically significant results, the posthoc power analysis showed that the statistical power (1 - *β*) exceeded 0.8 (i.e., critical value). These results indicated that the number of subjects in the present study was sufficient for the statistical procedures to detect statistically significant differences [38].
